# Rhizobacteria Impact Colonization of Listeria monocytogenes on Arabidopsis thaliana Roots

**DOI:** 10.1128/AEM.01411-21

**Published:** 2021-11-10

**Authors:** Alexi A. Schoenborn, Haley Clapper, Noam Eckshtain-Levi, Elizabeth A. Shank

**Affiliations:** a Department of Biology, University of North Carolina at Chapel Hill, Chapel Hill, North Carolina, USA; b Department of Microbiology and Immunology, University of North Carolina at Chapel Hill, Chapel Hill, North Carolina, USA; c Department of Systems Biology, University of Massachusetts Chan Medical School, Worcester, Massachusetts, USA; INRS—Institut Armand-Frappier

**Keywords:** *Listeria*, *Listeria monocytogenes*, food-borne pathogens, hydroponics, plant-microbe interactions

## Abstract

In spite of its relevance as a foodborne pathogen, we have limited knowledge about Listeria monocytogenes in the environment. L. monocytogenes outbreaks have been linked to fruits and vegetables; thus, a better understanding of the factors influencing its ability to colonize plants is important. We tested how environmental factors and other soil- and plant-associated bacteria influenced L. monocytogenes’ ability to colonize plant roots using Arabidopsis thaliana seedlings in a hydroponic growth system. We determined that the successful root colonization of L. monocytogenes 10403S was modestly but significantly enhanced by the bacterium being pregrown at higher temperatures, and this effect was independent of the biofilm and virulence regulator PrfA. We tested 14 rhizosphere-derived bacteria for their impact on L. monocytogenes 10403S, identifying one that enhanced and 10 that inhibited the association of 10403S with plant roots. We also characterized the outcomes of these interactions under both coinoculation and invasion conditions. We characterized the physical requirements of five of these rhizobacteria to impact the association of L. monocytogenes 10403S with roots, visualizing one of these interactions by microscopy. Furthermore, we determined that two rhizobacteria (one an inhibitor, the other an enhancer of 10403S root association) were able to similarly impact 10 different L. monocytogenes strains, indicating that the effects of these rhizobacteria on L. monocytogenes are not strain specific. Taken together, our results advance our understanding of the parameters that affect L. monocytogenes plant root colonization, knowledge that may enable us to deter its association with and, thus, downstream contamination of, food crops.

**IMPORTANCE** Listeria monocytogenes is ubiquitous in the environment, being found in or on soil, water, plants, and wildlife. However, little is known about the requirements for L. monocytogenes’ existence in these settings. Recent L. monocytogenes outbreaks have been associated with contaminated produce; thus, we used a plant colonization model to investigate factors that alter L. monocytogenes’ ability to colonize plant roots. We show that L. monocytogenes colonization of roots was enhanced when grown at higher temperatures prior to inoculation but did not require a known regulator of virulence and biofilm formation. Additionally, we identified several rhizobacteria that altered the ability of 11 different strains of L. monocytogenes to colonize plant roots. Understanding the factors that impact L. monocytogenes physiology and growth will be crucial for finding mechanisms (whether chemical or microbial) that enable its removal from plant surfaces to reduce L. monocytogenes contamination of produce and eliminate foodborne illness.

## INTRODUCTION

Listeria monocytogenes is a foodborne pathogen that predominantly infects immunocompromised individuals and causes the disease listeriosis (1). Although the chances of becoming infected with L. monocytogenes are low, the mortality rate for infection is roughly 30%, one of the highest rates for foodborne illnesses (1). Historically, L. monocytogenes has been implicated in food recalls associated with contaminated meat, fish, and dairy products. Recently, however, many food recalls and major outbreaks involving L. monocytogenes have been associated with contaminated fruits and vegetables (2).

L. monocytogenes has the potential to contaminate produce at several points along the food’s journey from the farm to the consumer (3–5). At the farm, fruits and vegetables are grown in close contact with soil (6), wild and domesticated animals (7), and diverse water sources (8), all of which have been suggested to be natural reservoirs for L. monocytogenes (6, 9–11). At the postharvest stage, food-processing facilities are another likely source of L. monocytogenes contamination (12–14). Several studies have demonstrated that L. monocytogenes can quickly adhere to and colonize an extensive range of produce, including leafy greens, sprouts, corn, alfalfa, melons, and celery but not carrots and tomatoes (11, 15–21). However, gaps still remain in our understanding of how environmental factors such as temperature and other rhizobacteria influence how L. monocytogenes colonizes roots.

In contrast to our incomplete understanding of the natural history of L. monocytogenes in environmentally relevant contexts related to plant colonization, there exists a vast body of knowledge about factors that impact L. monocytogenes associations with mammalian hosts (1, 22, 23). L. monocytogenes can survive and grow at a wide range of temperatures, spanning from 0 to 45°C (24). Many virulence genes, including the master virulence regulator, PrfA, have enhanced expression at elevated temperatures (e.g., 37°C) (25–28), while genes involved in chemotaxis and flagella are expressed at lower temperatures (e.g., 10 to 25°C) (29, 30). This division of gene regulation at different temperatures is likely what allows L. monocytogenes to adapt to a range of environments. Studies exploring the impact of temperature on L. monocytogenes’ ability to colonize surfaces found that biofilm formation was generally enhanced at higher temperatures (31, 32). Thus, many of the genes important for pathogenesis and virulence of L. monocytogenes may also impact physiological behaviors important during plant colonization. For instance, *actA*, a PrfA-regulated virulence factor, is important for L. monocytogenes aggregation (33), a possible mode of adhering to plant roots, and *flaA* (the gene encoding flagellin) influences plant colonization of L. monocytogenes in a bacterial strain- and plant-dependent manner (34). However, it remains unknown whether any of these genes are relevant to the ability of L. monocytogenes to colonize plant roots.

Another environmental variable that has the potential to alter L. monocytogenes colonization of plants is the resident soil and plant microbial community. Plant roots and the rhizosphere (the soil in close physical association with plant roots) harbor a diverse microbial community (35). There are different reports on how soil and plant microbial communities affect L. monocytogenes. Several studies have shown that a more diverse microbial community is inhibitory toward L. monocytogenes growth (6, 36, 37), while others have shown that diversity is not inhibitory (38). These disparate results indicate that the diversity of organisms alone is not a predictor of how microbes impact L. monocytogenes growth; instead, they suggest that specific microorganisms present within these communities are exerting defined impacts on L. monocytogenes (39).

Obtaining a better understanding of the environmental factors that affect L. monocytogenes associations with plants, particularly temperature and rhizobacteria, will have important implications for our understanding of the environmental lifestyle of L. monocytogenes as well as for suggesting potential means to mitigate its transmission into the food supply. We used our previously developed hydroponic growth system (14, 40) to monitor the association of Listeria monocytogenes 10403S (a streptomycin-resistant variant here referred to simply as 10403S) with *A. thaliana* seedling roots. This assay uses floating mesh to keep the leaves of *A. thaliana* seedlings above the liquid (which decreases plant stress) while allowing the roots to free-float in the liquid medium below (14). The advantage of this hydroponic system is that it allows for easy and reproducible manipulation of growth conditions and the addition of bacteria into the liquid medium where the roots are growing (41). It also enables numbers of bacterial CFU to be determined both from the liquid (as planktonic cells) and from the plant root (monitored after mild sonication to disassociate them from the root) (14). We chose to use L. monocytogenes strain 10403S because its resistance to streptomycin allowed us to select and quantify 10403S cell numbers when coinoculated with other rhizobacteria. We then investigated the ability of multiple different strains of L. monocytogenes to colonize *A. thaliana* roots in coculture with other rhizobacteria. We identified both bacteria with beneficial or antagonistic outcomes on L. monocytogenes plant colonization and characterized the sequential and physical requirements of these interactions.

## RESULTS

### Listeria monocytogenes readily colonizes Arabidopsis thaliana seedlings and persists on roots for 72 h.

**FIG 1:**
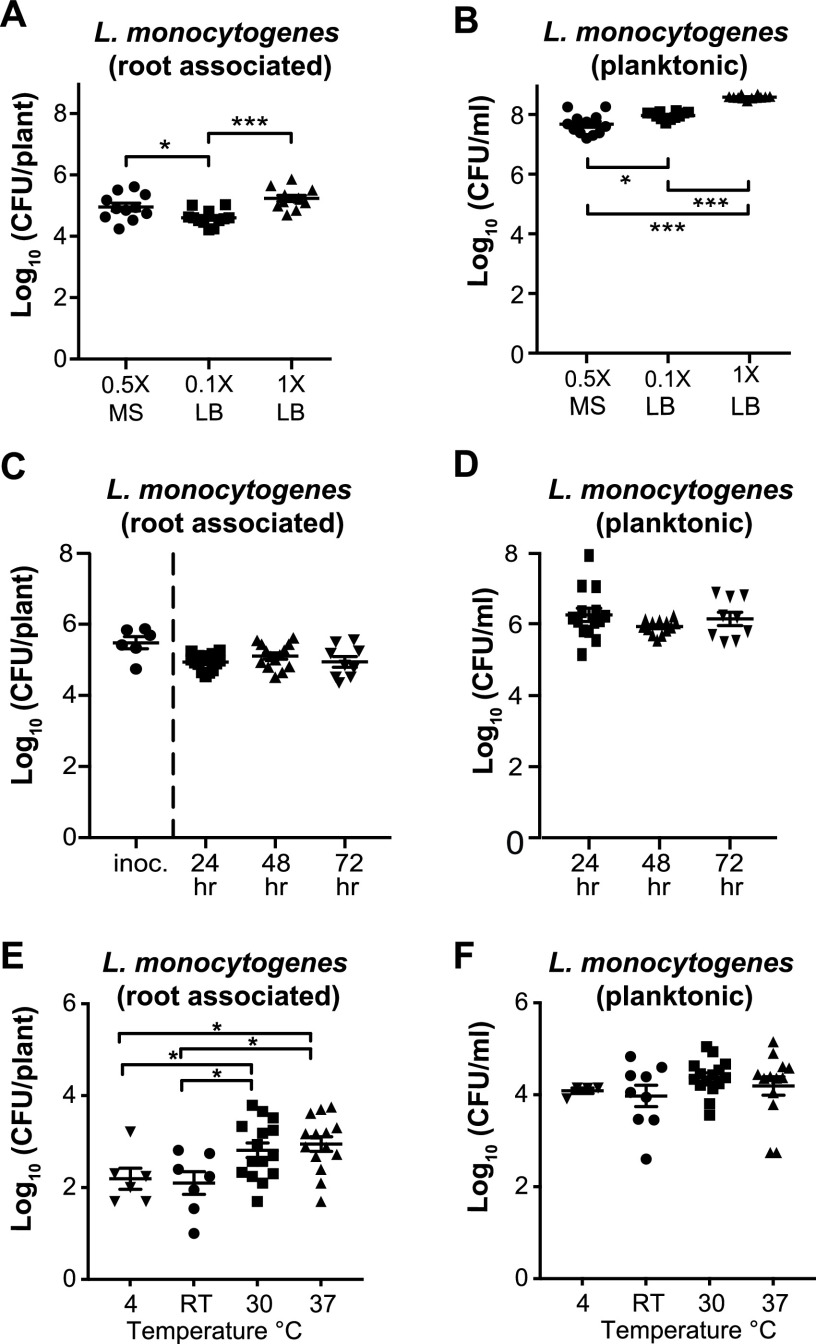
Establishing hydroponic root colonization assay conditions for L. monocytogenes. (A and B) Hydroponic colonization of *A. thaliana* roots by L. monocytogenes 10403S was performed in 0.5× Murashige and Skoog (MS), 0.1× LB, and 1× LB liquid medium. After incubating at RT for 24 h, seedlings were removed from the mesh floats and homogenized to determine number of CFU/plant (A) and CFU/ml liquid (B) based on serial dilutions (in all cases, seedlings were sonicated). (C and D) *A. thaliana* roots inoculated with 10403S at ∼10^5^ CFU/plant were transferred to fresh 0.5× MS medium and incubated for 24, 48, or 72 h. After incubation, seedlings were removed from mesh floats and homogenized to determine number of CFU/plant (C) and CFU/ml liquid (D); no significant differences were observed. (E and F) L. monocytogenes pregrown at 4°C, RT (20 to 22°C), 30°C, or 37°C prior to inoculation was added to the hydroponic assay using 0.5× MS and incubated at RT for 24 h, after which numbers of CFU/plant (E) and CFU/ml liquid (F) were determined. Kruskal-Wallis ANOVA and Mann-Whitney *t* test were used for statistical comparisons. Asterisks denote *P* values (*, *P < *0.05; **, *P < *0.01; ***, *P < *0.001).

To establish hydroponic assay conditions, we compared the ability of L. monocytogenes 10403S to colonize *A. thaliana* roots in two growth media at different concentrations: lysogeny broth (LB) (1× and 0.1×) and Murashige and Skoog (0.5× MS). After a 24-h incubation, 10403S was similarly abundant on *A. thaliana* seedlings in all media (∼10^4^ to 10^5^ CFU/plant) (Fig. 1A). However, the smallest numbers of 10403S planktonic cells were seen in 0.5× MS (∼10^7^ CFU/ml) compared to the LB media (∼10^8^ to 10^9^ CFU/ml), indicating that 10403S more specifically associated with the roots in 0.5× MS. Because of this, we elected to use 0.5× MS in all subsequent assays.

We next wanted to investigate if 10403S was able to persist over time on *A. thaliana* roots in 0.5× MS. To test persistence, we first preinoculated *A. thaliana* roots with 10403S to an attachment level of ∼10^6^ CFU/plant (Fig. 1C [innoc]). After 3 h, we transferred the inoculated seedlings to fresh 0.5× MS medium and determined the number of 10403S cells present on the root and as planktonic cells per well after 24, 48, and 72 h. At 24 h postinoculation, the colonization numbers fell slightly from 10^6^ CFU/plant at inoculation to 10^5^ CFU/plant; this number of cells was then maintained from 24 to 72 h (Fig. 1C). Additionally, the number of planktonic cells did not change significantly during this time course (remaining at ∼10^6^ CFU/ml) (Fig. 1D). This suggests that L. monocytogenes 10403S can persist on plant roots for at least 3 days under these conditions.

### Pregrowth at 30°C and 37°C before inoculation enhanced 10403S root colonization.

L. monocytogenes can grow under a range of temperatures, 0 to 45°C, and growth temperature can significantly alter its transcriptional, translational, and metabolic state (25, 26, 28, 42, 43). We therefore wanted to determine whether 10403S being pregrown at different temperatures (before inoculation into the hydroponic assay) impacted the bacterium’s ability to colonize roots. To test this, we grew 10403S at 4°C, room temperature (RT), 30°C, and 37°C and used optical density at 600 nm (OD_600_)-normalized dilutions to inoculate our hydroponic assay, after which the seedlings were incubated at RT and number of CFU/plant was assessed after 24 h.

10403S pregrown at 30°C and 37°C had modest but significantly increased number of CFU/plant (∼10^3^ CFU/plant) compared to bacteria pregrown at 4°C or RT (∼3 × 10^2^ CFU/plant) (Fig. 1E), with no differences in planktonic number of CFU/plant across temperature conditions (all ∼10^4^ CFU/ml) (Fig. 1F). As a control, we inoculated 10403S grown at different preinoculation temperatures into 0.5× MS without a plant seedling and did not observe any significant differences between number of CFU/plant at different temperatures (see Fig. S1 in the supplemental material). This indicates that the bacteria pregrown at higher temperatures were more effective at colonizing the plant root despite reaching the same overall planktonic cell numbers.

### Colonization of L. monocytogenes 10403S does not require *prfA*, *flaA*, or *actA*.

Based on the response of 10403S to growth temperature, we speculated that the biofilm and virulence regulator PrfA are involved in root colonization of 10403S. To determine this, we compared the root colonization of the 10403S parental strain to strains either lacking the *prfA* gene or constitutively expressing *prfA* (prfA*). All strains were grown at 37°C (preinoculation), and after 24 h, we compared the plant-associated and planktonic CFU numbers between the three L. monocytogenes strains. There were no significant differences in either number of CFU/plant (all ∼10^4^ CFU/plant) (Fig. S2A) or number of CFU/ml planktonic (all ∼10^6^ CFU/ml) (Fig. S2B). To further validate these findings, we performed an alternative root colonization assay that used a modified hydroponic approach involving higher inoculation levels but reduced colonization time. Even with these altered conditions, we did not observe any significant differences in number of CFU/plant between the Δ*prfA*, prfA*, and WT strains (all ∼10^5^ CFU/plant) (Fig. S2C). We also tested (using our alternative hydroponic assay) whether *flaA* and *actA* impacted L. monocytogenes’ ability to colonize roots at elevated temperatures and saw colonization levels similar to those of the parental strain. These data support the conclusion that L. monocytogenes 10403S root colonization is not regulated by PrfA, *flaA*, or *actA.*

### Coinoculation with rhizosphere bacteria impacts L. monocytogenes 10403S plant colonization.

Next, we investigated whether we could identify other environmental factors that influenced L. monocytogenes’ ability to colonize *A. thaliana* roots. Plant roots in native soil harbor diverse microbial communities (35); we therefore examined whether other bacteria impacted 10403S root association. Previous work has identified several rhizobacteria that are strong hydroponic colonizers of *A. thaliana* roots: Arthrobacter nicotinovorans (ES1024), *Curtobacterium oceanosedimentum* (ES1096), Microbacterium oleivorans (ES1039), and Pseudomonas simiae WCS417r (ES620) (14). We tested the effects of these microbes in dual-species coculture with 10403S on *A. thaliana* roots. For these experiments, we added equivalent levels (based on OD_600_) of 10403S and its coinoculation partner within the same liquid well and then assayed for 10403S colonization after 24 h using streptomycin to select for 10403S. To verify that the rhizobacteria were still colonizing the root and present in the liquid media, we also assessed the number of CFU/plant (Fig. S3A) and number of CFU/ml of the rhizobacteria in monoculture with the plant and when coinoculated with 10403S (Fig. S3B).

**FIG 2:**
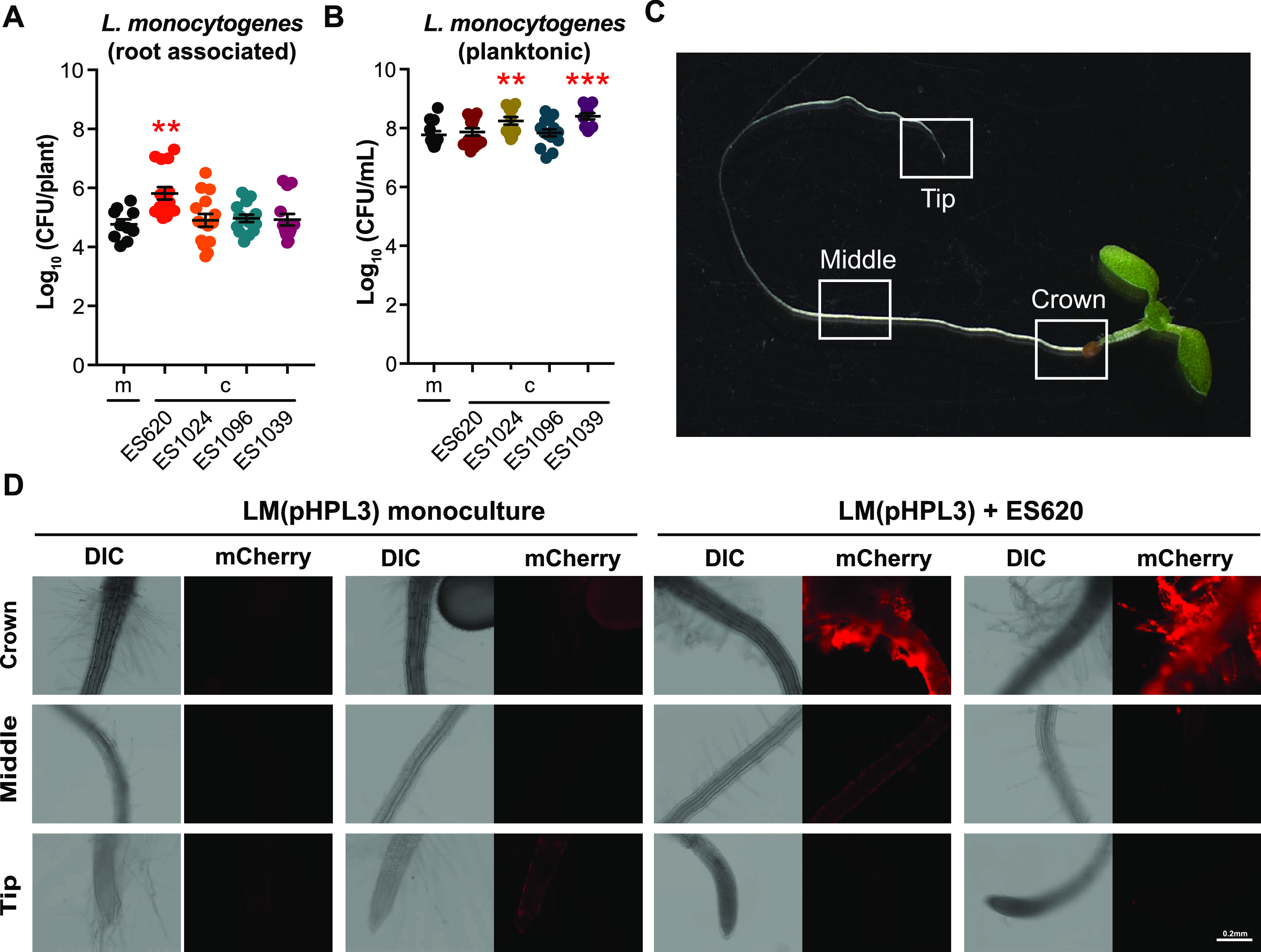
Pseudomonas simiae (ES620) increases L. monocytogenes 10403S root colonization. Arthrobacter nicotinovorans (ES1014), *Curtobacterium oceanosedimentum* (ES1096), Microbacterium oleivorans (ES1039), and Pseudomonas simiae WCS417r (ES620) were cocultured with L. monocytogenes 10403S for 24 h at RT. After incubation, seedlings were removed from the wells, homogenized, and serial dilutions plated on 1× LB with streptomycin to calculate the number of CFU/plant (A) and CFU/ml liquid (B) L. monocytogenes 10403S in monoculture (m) or coculture (c). Statistics were performed using Kruskal-Wallis ANOVA and Mann-Whitney *t* tests comparing coculture to monoculture CFU. Asterisks denote *P* value (**, *P < *0.01; ***, *P < *0.001). (C) *A. thaliana*, 9 days after germination, imaged at ×8 magnification to show crown, middle, and root tip regions, where differential interference contrast and fluorescent images were obtained. (D) Distribution of L. monocytogenes 10403S(pHPL3) on seedling roots in monoculture or coculture with ES620. Fluorescent cells were false-colored red (mCherry). Representative images from two different seedlings are shown. Scale bar, 0.2 mm.

We discovered that when 10430S was coinoculated with ES1014, ES1096, or ES1039, 10403S had a similar number of CFU/plant as when inoculated in monoculture (∼10^5^ CFU/plant) (Fig. 2A). However, the number of CFU/plant of 10403S significantly increased (*P < *0.05) when it was coinoculated with ES620 (to ∼10^6^ CFU/plant) (Fig. 2A). We also assessed the number of CFU of planktonic 10403S in each well and discovered that, when coinoculated with ES1024 or ES1039, 10403S was present at significantly higher (*P < *0.05) numbers of CFU/ml than when grown alone (5 × 10^8^ versus 1 × 10^8^ CFU/ml, respectively) (Fig. 2B). However, there was no significant difference in planktonic number of CFU/ml when 10403S was coinoculated with ES620 or ES1039 (Fig. 2B). Thus, ES1039 (*M. oleivorans*) does not appear to affect the growth of 10403S in liquid or on roots, while ES1024 (*A. nicotinovorans*) and ES1096 (*C. oceanosedimentum*) appeared to stimulate 10403S planktonic growth without altering its levels on roots.

### The ability of ES620 (*P. simiae*) to positively impact L. monocytogenes 10403S plant colonization appears contact dependent.

We then wanted to determine if the physical presence of ES620 was essential for enhancing the root association of L. monocytogenes 10403S. To address this, we collected cell-free, conditioned medium (CM) from ES620 and 10403S when grown alone. We then preinoculated seedlings with 10403S and added these seedlings to wells containing CM from either 10403S or ES620 (1 part fresh 0.5× MS to 1 part CM). After 24 h, we did not detect a significant difference in root-associated 10403S number of CFU/plant (Fig. S4A) when grown in CM from itself versus from ES620 (both ∼10^5^ CFU/plant). However, we did detect a significant increase (*P < *0.05 with ∼2-log increase) in the number of CFU/ml of 10403S planktonic cells when grown in ES620 CM compared to 10403S CM (Fig. S4B). These data indicate that the enhanced root association of 10403S observed when coinoculated with ES620 requires the physical presence of ES620.

We next wanted to determine if the presence of ES620 (*P. simiae*) altered colonization patterns or localization of 10403S on the root. To do this, we conjugated Escherichia coli SM10 carrying the constitutively fluorescent plasmid, pHPL3-mCherry (Cm^r^), with 10403S to generate a strain we called 10403S(pHPL3). 10403S(pHPL3) was cultured in the hydroponic assay in monoculture or in coculture with ES620. ES620 did not autofluoresce in the mCherry channel (Fig. S5). We targeted three main areas of the seedling root for imaging: the crown, middle, and root tip (Fig. 2C). When grown in monoculture with the *A. thaliana* seedling, 10403S(pHPL3) appears to colonize all three regions we assessed, with the most colonization appearing in the middle (Fig. 2D). When coinoculated with ES620, we observed an increase in overall L. monocytogenes fluorescence on the root, particularly at the crown and middle sections and along the root hairs (Fig. 2D, Fig. S5). Overall, these images demonstrate that 10403S(pHPL3) is a robust root colonizer even when in competition with other bacteria proficient at colonizing roots, and that these coculture interactions may alter the localization patterns of 10403S(pHPL3) along the root.

### Ten additional L. monocytogenes strains, encompassing a range of serotypes, have enhanced root colonization when coinoculated with ES620.

We next wanted to determine whether ES620 could affect the root association of other L. monocytogenes strains. We obtained 10 L. monocytogenes strains (Table 1) representing a wide array of serotypes and lineages and that can colonize alfalfa sprouts (20). To begin, we tested the root colonization of these L. monocytogenes strains in monoculture. All 10 strains colonized *A. thaliana* seedling roots to ∼10^4^ to 10^5^ CFU/plant (Fig. S6A). Four strains (RM3171, RM3169, RM3000, and RM2999) had significantly reduced numbers of CFU/plant, and two strains (RM3169 and RM3000) had significantly reduced planktonic numbers of CFU/ml in monoculture compared to 10403S (*P < *0.05) (Fig. S6B). Overall, these data demonstrate that all 10 of these additional L. monocytogenes strains could colonize *A. thaliana* to substantial levels.

**TABLE 1:** Bacterial strains used

Strain ID	Species	Reference or source
10403S	Listeria monocytogenes streptomycin-resistant variant (parental strain) (serotype 1/2a)	87
DP-L4650, ES959	10403S Δ*flaA*	88
DP-L3078, ES949	10403S Δ*actA*	89
RM3171	L. monocytogenes (serotype 4a)	20
RM3169	L. monocytogenes (serotype 4a, 4c)	20
RM3160	L. monocytogenes (serotype 1/2a)	20
RM3153	L. monocytogenes (serotype 4b)	20
RM3100	L. monocytogenes (serotype 4b)	20
RM3000	L. monocytogenes (serotype 1/2c)	20
RM2999	L. monocytogenes (serotype 4b)	20
RM2992	L. monocytogenes (serotype 4b, 4d/4e)	20
RM2388	L. monocytogenes (serotype 1/2a)	20
RM2387	L. monocytogenes (serotype 4b)	20
DP-L4317	10403S Δ*prfA*: in-frame deletion (amino acids 34–146)	90
NF-L1177	10403S PrfA* [encoded by *prfA*(G145S)]	91
ES558	Pseudomonas fluorescens Pf-5	ATCC BAA-477
ES1007	Pseudomonas fluorescens BZ64	44, 92
ES1010	Burkholderia cenocepacia 6	44, 92
ES1016	Pseudomonas umsongensis 20MGCvi1.1	44, 92
ES1026	Pseudomonas sp. strain 35MFCvi1.1	44, 92
ES1027	Pseudomonas mandelii 36MFCvi1.1	44, 92
ES1030	Pseudomonas sp. strain 45MFCvi1.1	44, 92
ES1032	Pseudomonas sp. strain 48MFCvi1.1	44, 92
ES1034	Pseudomonas sp. strain KD5	44, 92
ES1035	Pseudomonas brassicacearum 51MFCvi2.1	44, 92
ES620	Pseudomonas simiae WCS417r	14, 93
ES1024	Arthrobacter nicotinovorans	14, 44, 92
ES1039	Microbacterium oleivorans	14, 44, 92
ES1096	*Curtobacterium oceanosedimentum*	14, 44, 92
DP-E6572	Conjugation; *thi-1 thr-1 leuB6 tonA21 lacY1 supE44 recA* λ^−^ integrated [RP4-2-Tcr∷Mu] *aphA*^+^ (Km^r^) Tra^+^ SM10 + pHPL3-mCherry (Cm^r^)	84, 94
ES2751	10403S + pHPL3-mCherry (Cm^r^)	This study

**FIG 3:**
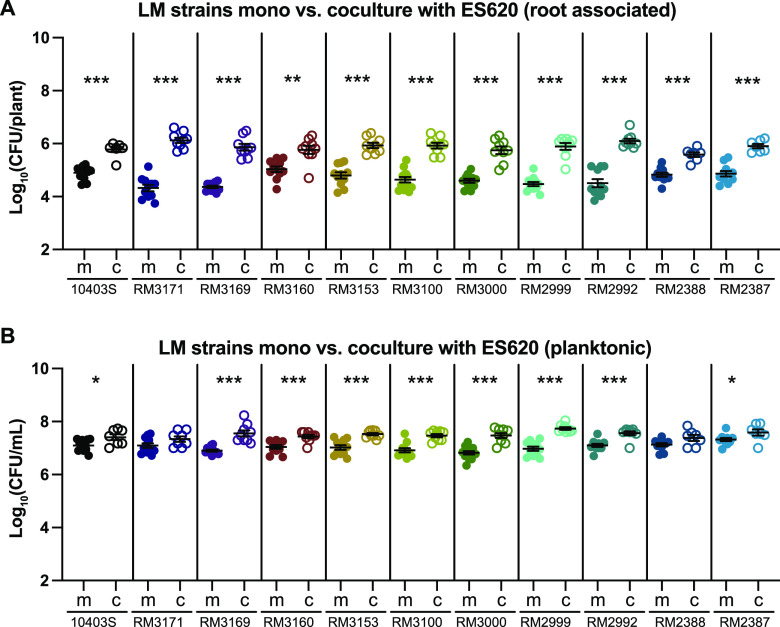
Ten additional L. monocytogenes strains, encompassing an array of serotypes and lineages, all have enhanced root colonization when cocultured with ES620 (*P. simiae*). L. monocytogenes strains were coinoculated with ES620 for 24 h at RT. After incubation, seedlings were removed from the wells, homogenized, and serial dilutions plated on 1× LB to calculate the number of CFU/plant (A) and CFU/ml liquid (B) L. monocytogenes in monoculture (m) or coculture (c). We distinguished between ES620 and L. monocytogenes by color and morphology. Statistics were performed using Kruskal-Wallis ANOVA and Mann-Whitney *t* tests comparing coculture to monoculture CFU. Asterisks denote *P* values (*, *P < *0.05; **, *P < *0.01; ***, *P < *0.001).

When we coinoculated these 10 L. monocytogenes strains with ES620 (*P. simiae*), all had significantly (*P < *0.05) enhanced root colonization compared to when inoculated alone (Fig. 3A). Four strains had an ∼1-log fold increase in CFU numbers when inoculated with ES620, while the other six increased ∼2 log when grown with ES620 compared to monoculture inoculation (Fig. 3A). We also assessed the planktonic number of CFU/ml of each L. monocytogenes strain inoculated alone or with ES620 and found a significantly enhanced number of CFU/ml when RM3169, RM3160, RM3153, RM3100, RM3000, RM2999, RM2992, and RM2387 (*P < *0.05) were coinoculated with ES620 (Fig. 3B). Additionally, we quantified ES620 in monoculture and coculture and primarily saw changes in CFU/ml planktonic cell numbers (Fig. S3C and D). Overall, these data suggest that the ability of ES620 to enhance root colonization of 10403S is not strain specific but instead that ES620 is broadly able to enhance the colonization of a wide range of L. monocytogenes strains on *A. thaliana* roots.

### Agar-based coculture to identify rhizobacteria antagonistic to 10403S.

Based on the range of observed outcomes when L. monocytogenes 10403S was coinoculated with different rhizobacteria, we next wanted to assess L. monocytogenes’ interactions with additional root-associated bacteria, with the goal of identifying bacteria that reduced L. monocytogenes’ association with plant roots. We utilized a bacterial collection containing 125 fully genome-sequenced isolates obtained from the rhizosphere of *A. thaliana* grown in soil (44). We initially screened these strains using an agar-based assay to identify bacteria with the ability to antagonize L. monocytogenes 10403S and then used our hydroponic assay to determine whether they specifically impacted L. monocytogenes’ association with plants.

**FIG 4:**
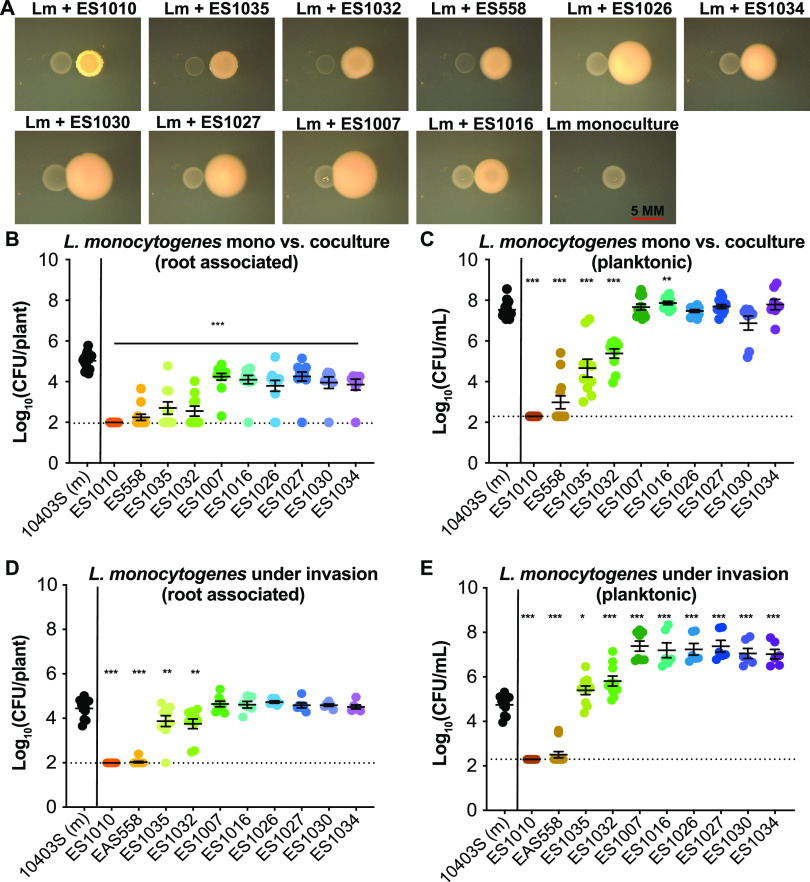
Coculture and invasion of rhizobacteria alter the levels of L. monocytogenes 10403S on plant roots. (A) Agar-based cocultures grown at 30°C for 48 h. In all panels, L. monocytogenes is the colony on the left with the other rhizobacteria being the colony on the right. Scale bar, 5 mm. (B and C) In the coculture hydroponic assay, L. monocytogenes 10403S was inoculated in monoculture (at an OD_600_ of 0.02) or in a 1:1 ratio with a rhizobacterium (each at OD_600_ of 0.02) with *A. thaliana* seedling roots for 24 h at RT. After incubation, seedlings were removed from the wells, homogenized, and serial dilutions plated on 1× LB with streptomycin to select for L. monocytogenes 10403S and determine the number of CFU/plant (B) and CFU/ml liquid (C). (D and E) In the invasion assay, L. monocytogenes 10403S was preloaded onto a seedling root (at ∼10^5^ CFU/plant) then transferred to wells with a single rhizobacterium at an OD_600_ of 0.02. After 24 h at RT, seedlings were removed, sonicated, and serially diluted on 1× LB with streptomycin to select for L. monocytogenes 10403S and determine number of CFU/plant (D) and CFU/ml liquid (E). Asterisks denote *P* values (*, *P < *0.05; **, *P < *0.01; ***, *P < *0.001). Dashed lines indicate level of detection for the assay. If samples were undetected they were assigned a value immediately below the level of detection.

We spotted cultures of either L. monocytogenes 10403S alone (in monoculture) or next to one of the 125 bacterial isolates (in coculture) onto agar plates. We identified 18 bacterial isolates, mostly represented by Pseudomonas and *Burkholderia* species, that demonstrated noticeable colony-level antagonism toward L. monocytogenes 10403S based on visual increases in translucence of L. monocytogenes 10403S colonies or a reduction in overall biomass (Fig. 4A, Table S1). We conducted additional assays with 10 out of the 18 isolates (one of the *Burkholderia* rhizosphere isolates, a subset of the Pseudomonas rhizosphere isolates, and a Pseudomonas type strain [Pf-5]).

### Rhizobacteria show divergent effects on 10403S root colonization.

We first ensured that all 10 rhizobacteria could colonize *A. thaliana* roots both when inoculated on their own or during coinoculation with L. monocytogenes 10403S (Fig. S3A); they all also had detectable planktonic numbers of CFU/ml in the wells (Fig. S3B). All 10 strains significantly reduced L. monocytogenes 10403S’ ability to colonize roots (*P* < 0.001) (Fig. 4B). ES1010, ES558, ES1035, and ES1032 had the most significant impact against L. monocytogenes 10403S, reducing its plant colonization from 10^5^ CFU/plant in monoculture to ∼10^3^ CFU/plant during coinoculation (Fig. 4B). These strains also significantly reduced L. monocytogenes planktonic cells from ∼10^7^ CFU/ml (monoculture) to ∼10^3^ CFU/ml (coculture) (Fig. 4C). This indicates that these strains exhibit generalized killing of L. monocytogenes whether coinoculated on the root or in liquid. Interestingly, these four rhizobacteria led to a 2-log decrease in the number of 10403S CFU/plant compared to a 4-log decrease in planktonic number of CFU/ml, implying that 10403S cells associated with the root are protected from killing by these rhizobacteria.

The other six rhizobacterial isolates examined (ES1007, ES1016, ES1026, ES1027, ES1030, and ES1034) all significantly inhibited 10403S root colonization (Fig. 4B) but did not negatively impact 10403S planktonic growth (Fig. 4C); in one case, the coculture partner (ES1016) even significantly enhanced (*P < *0.05) 10403S growth in the liquid (Fig. 4B and C). These data suggest that these strains are outcompeting or specifically excluding 10403S from the plant root. These results also indicate that multiple pseudomonads can reduce 10403S attachment to seedling roots during coculture as well as demonstrating the importance of strain-level variability of different bacteria to either help or hinder 10403S colonization of plant roots.

### Roots preinoculated with L. monocytogenes 10403S are susceptible to antagonism by rhizobacteria.

We next wanted to determine whether the order of addition of bacteria to roots altered the outcome concerning the number of L. monocytogenes CFU/plant to determine whether we could identify bacteria able to inhibit L. monocytogenes even after it was established on roots. We modified the assay to mimic an invasion scenario, where 10403S was preinoculated on roots (to ∼10^5^ CFU/ml) before being moved to wells containing individual rhizobacteria (rather than inoculating the two strains simultaneously). After 24 h, we compared the number of 10403S CFU/plant under rhizobacterial invasion to its number of CFU/plant in monoculture without invasion. The rhizobacterial strains able to reduce 10403S CFU both on and off the root during coinoculation (ES1010, ES558, ES1035, and ES1032) also significantly reduced (*P < *0.05) the number of 10403S CFU/plant when added as invaders (Fig. 4D). ES1010 and ES558 had the strongest levels of antagonism, reducing 10403S on the root by ∼3 log (Fig. 4D). ES1035 and ES1032 reduced the level of 10403S by about a log (from 10^5^ to 10^4^ CFU/plant) (Fig. 4D). Notably, ES1010 and ES558 also drastically reduced (*P < *0.05) the number of 10403S CFU/ml in the media (from ∼10^5^ CFU/ml when 10403S was grown without an invader to <10^3^ CFU/ml [below the level of detection]) (Fig. 4E). In contrast, the other rhizobacteria (ES1007, ES1016, ES1026, ES1027, ES1030, and ES1034) were not detrimental to number of 10403S CFU/plant when added as invaders (Fig. 4D) and increased 10403S from 10^5^ to 10^7^ CFU/ml in the wells (Fig. 4E).

To determine whether the inability of these rhizobacteria to invade 10403S on plant roots was because 10403S simply excluded them from accessing the root, we quantified the number of CFU of the invading bacteria both on the plant and in the liquid culture medium. We found that all rhizobacterial strains could colonize the plants to ∼10^5^ CFU/plant or greater even as invaders and were present in the media at levels near 10^8^ CFU/ml (Fig. S7). Collectively, these data highlight the importance of investigating the sequential colonization of bacterial consortium members: several bacteria (ES1007, ES1016, ES1026, ES1027, ES1030, and ES1034) that were antagonistic when added concurrently with 10403S during colonization were unable to impact 10403S cell numbers on the root if it had already become established there.

### Many rhizobacteria inhibit the colonization of roots by 10403S via secreted compounds.

Having found that physical contact was required for the enhanced root colonization observed when 10403S was coinoculated with ES620, we next wondered whether physical contact was also necessary for the negative impact that ES1010, ES558, ES1032, and ES1035 had on root colonization by 10403S. To test this, we obtained CM from these strains grown in monoculture with *A. thaliana* roots. We placed seedlings containing 10403S (∼10^5^ CFU/plant) in wells containing 1:1 fresh 0.5× MS-CM and assessed 10403S number of CFU/plant and number of CFU/ml liquid after 24 h. 10403S grown in CM of ES558, ES1010, ES1032, or ES1035 had significantly reduced (*P < *0.05) number of CFU/plant compared to when it was grown in 10403S-CM (Fig. S8A). The CM from ES558 led to the greatest decrease in root-associated 10403S (dropping colonization from 10^5^ to 10^4^ CFU/plant), while the other isolates reduced 10403S colonization by a half log (Fig. S8A). The CM of these four strains had divergent impacts on 10403S growth in the hydroponic liquid: number of 10403S CFU/ml was significantly enhanced (*P < *0.05) by the CM from ES1010, reduced by the CM from ES558 and ES1035 (*P < *0.05), and unaffected by the CM of ES1032 (Fig. S8B). This suggested that the antagonistic effects of these strains toward L. monocytogenes 10403S root association is at least partially mediated by secreted chemical cues.

### CM from ES558 (P. protegens Pf-5) significantly reduced the plant association and planktonic CFU of all L. monocytogenes strains tested.

**FIG 5:**
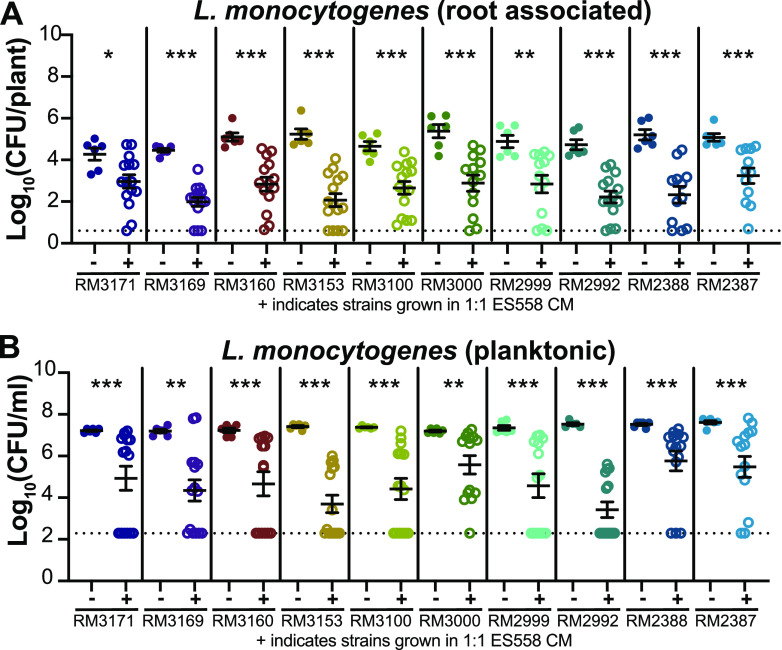
Conditioned medium from Pseudomonas protegens (ES558) reduced root association and liquid survival of 10 additional L. monocytogenes strains. Conditioned medium (CM) was collected by centrifuging and filter sterilizing the liquid from bacteria grown with hydroponic plants after 24 h of incubation at RT and then used fresh. CM from either L. monocytogenes strains (as a control) or ES558 were mixed at a 1:1 concentration (0.5× fresh MS to 0.5× CM) in the assay. L. monocytogenes numbers of CFU/plant (A) and CFU/ml (B) were determined by serial dilution on 1× LB. Statistics were performed using Mann-Whitney *t* tests comparing number of CFU of L. monocytogenes grown in its own CM to L. monocytogenes grown in ES558 CM. Asterisks denote *P* values (*, *P < *0.05; **, *P < *0.01; ***, *P < *0.001). Dashed lines indicate the level of detection. If samples were undetected, they were assigned a value immediately below the level of detection.

Having found that conditioned media from ES558, ES1010, ES1032, and ES1035 reduced the association of L. monocytogenes 10403S with roots, we next wanted to determine whether the CM from these rhizobacteria were also antagonistic toward other L. monocytogenes strains. Visual indicators of antagonism during agar-based cocultures demonstrated that all four rhizobacteria antagonized the additional L. monocytogenes strains, with ES558 (P. protegens) appearing to be the strongest antagonizer (Fig. S9). We therefore tested whether the CM from ES558 could inhibit the other L. monocytogenes strains during plant inoculation. When we compared the 10 L. monocytogenes strains grown in either CM from themselves (−) or from ES558 (+), we observed a significant decrease (*P < *0.05) in root-associated L. monocytogenes CFU number (Fig. 5A) and planktonic number of CFU/ml (Fig. 5B) for all L. monocytogenes strains investigated. This suggests that a secreted product produced by ES558 is sufficient to significantly reduce L. monocytogenes cell numbers on *A. thaliana* seedling roots.

## DISCUSSION

Current evidence has demonstrated that L. monocytogenes is prevalent in the environment and can contaminate produce and crops (3–5). While much of L. monocytogenes’ contamination of ready-to-eat foods has been linked to contaminated food-processing facilities and equipment (45, 46), it remains unclear whether low-level preharvest contamination of produce has the potential to play a role in facility-wide contamination problems (47). Studies investigating L. monocytogenes’ associations with plants *in vitro* have shown that L. monocytogenes can quickly colonize, persist, and grow on a wide variety of food produce (18). Our hydroponic studies support these observations. We demonstrate that L. monocytogenes 10403S, as well as 10 other L. monocytogenes strains encompassing a wide array of L. monocytogenes lineages and serotypes, can quickly and robustly colonize *A. thaliana* roots. In addition, although many studies have investigated the factors that impact L. monocytogenes’ ability to colonize mammalian hosts, little is known about the environmental variables that influence L. monocytogenes’ ability to robustly colonize plants and their roots. Here, we demonstrate that preinoculation growth temperature influences L. monocytogenes 10403S root colonization in a PrfA-independent manner. Additionally, we showed that specific plant-associated rhizobacteria can enhance or inhibit L. monocytogenes’ ability to colonize *A. thaliana* roots. Depending on the particular bacterial species interacting with L. monocytogenes 10403S, the sequence of colonization and the physical presence of bacteria on roots both appear to be important determinants of colonization outcome.

L. monocytogenes can survive and grow in a wide range of temperatures, including low temperatures such as those used during refrigeration (48). This is problematic in the food-processing industry, where low temperatures are typically used to preserve food and prevent bacterial growth. Previous studies investigating the impacts of temperature on L. monocytogenes biofilm formation have demonstrated that incubations at higher temperatures often yield enhanced biofilms on abiotic surfaces (31, 49). Our work corroborates these findings and further suggests that temperature-induced changes can persist and alter phenotypes (such as the ability to colonize roots) over extended periods of time. This plasticity is likely due to L. monocytogenes’ ability to dramatically alter its transcriptional state (25, 26, 28) and metabolome and proteome when subjected to a range of temperatures (42, 50).

Exposure to stressors, such as temperature, pH, limited nutrients, and other physiochemical stressors, has been found to enhance L. monocytogenes’ survival in the soil and other environmental conditions (36, 51–53). One stress response regulator activated by these stressors includes the major virulence regulator PrfA. PrfA impacts biofilm formation of L. monocytogenes on abiotic surfaces (54) as well as being important for survival in the soil (55). In addition, the PrfA mRNA transcript contains a thermosensor (25, 26, 41). These data raised the possibility that PrfA explains the temperature effect we observed in L. monocytogenes 10403S associations with plant roots. However, consistent with other studies (55, 56), we saw that PrfA was not essential for *A. thaliana* root colonization (see Fig. S2A in the supplemental material). Additionally, we did not see any colonization defects when we assessed the *flaA* and *actA* mutants (Fig. S2C). This indicates that there are still-unidentified factors regulating the surface association of L. monocytogenes and that some of these pathways are affected by temperature. Other possible gene candidates that may be influencing these outcomes are the alternative sigma factor B (*sigB*) or the response regulator AgrA, both of which are influential in environmental survival and adaptation in L. monocytogenes, as well as playing roles in biofilm/plant colonization (56, 57). Future work investigating such candidate genes using plant model systems as well as screening a library of L. monocytogenes transposon mutants for their ability to colonize roots could be conducted to identify genes involved in plant colonization. We speculate that such efforts could lead to the discovery of genes that regulate the expression of biofilm-like genes involved in L. monocytogenes root association (and potentially to other biotic surfaces) as well as enable the identification of genes specifically responsive to temperature. This idea is particularly intriguing given the evidence that L. monocytogenes does not form robust biofilms on stainless steel coupons or other abiotic surfaces typically used during *in vitro* assays (58). Such poor biofilm formation is in direct contrast to our findings that L. monocytogenes quickly colonizes roots to CFU/plant levels that are similar to or better than those of other well-known rhizobacteria, such as *P. simiae* (ES620). Altogether, these data suggest that L. monocytogenes forms more robust biofilms on biotic surfaces than on abiotic surfaces and indicate that the genetic factors governing the association of L. monocytogenes with roots remain to be discovered.

Biofilms that exist in nature (e.g., in soil and on roots) typically contain multiple species of bacteria (59, 60) that experience a wide range of interactions, from synergism and cooperation to competition or antagonism (61–64). Several interspecies interactions have been identified that alter L. monocytogenes’ ability to colonize food-processing-facility-like surfaces and dairy food items (14, 65). In addition, studies have identified bacteria that either enhance the growth of (66, 67) or are antagonistic toward (39, 65) L. monocytogenes during coculture on abiotic surfaces. In addition, Bacillus amyloliquefaciens inhibits L. monocytogenes growth on melons, results that highlight the need for further investigation into bacterial interactions that influence L. monocytogenes on plants (68). In our investigation of how rhizobacteria impact L. monocytogenes colonization of *A. thaliana* roots, we found that L. monocytogenes 10403S readily cocolonizes plant roots with an array of rhizobacteria (*A. nicotinovorans*, *C. oceanosedimentum*, and *M. oleivorans*, among others). Furthermore, when colonized with *P. simiae* WCS417r (ES620), 11 different L. monocytogenes strains, representing a range of serotypes and lineages, all exhibited enhanced root colonization compared to when inoculated on roots in monoculture. This particular interaction was dependent on the physical presence of ES620, since conditioned medium from this strain could not enhance L. monocytogenes 10403S colonization. These synergistic observations are consistent with studies describing how Pseudomonas fluorescens enhances L. monocytogenes attachment to glass coverslips and stainless steel (69). In additional studies, the presence of L. monocytogenes enhanced P. fluorescens’ biofilm matrix production, which protected both bacteria from external stressors such as disinfectants (70, 71). It is possible that similar mechanisms are at work between L. monocytogenes and the closely related bacterium *P. simiae* in our assay.

L. monocytogenes 10403S’ ability to coexist with our initial panel of four rhizobacteria was intriguing, as much of the literature often highlights antagonistic outcomes (72, 73); however, neutral and beneficial outcomes are also frequently observed (39). Of the 125 rhizobacteria we tested in adjacent agar coculture with L. monocytogenes, only 18 had visible signs of antagonism toward 10403S. Interestingly, however, seven of these antagonistic bacteria were Pseudomonas species, a relatively high proportion of the antagonists. Pseudomonas species are widely prevalent in the environment as well as in food-processing facilities (67, 74) and, thus, have a reasonable possibility of interacting with L. monocytogenes in these settings. That said, one recent study of tree fruit-processing facilities found 100% of the samples tested positive for L. monocytogenes at one of the sites. This same site also had a high abundance of *Pseudomonadaceae*, indicating that high levels of coexistence are also possible (67). Indeed, not all of the pseudomonads we screened were antagonistic toward 10403S, with five other Pseudomonas strains showing no impact on 10403S growth. In addition, *P. simiae* WCS417r (ES620) enhanced L. monocytogenes’ root attachment. Thus, even within bacterial genera with strong impacts on L. monocytogenes, a wide range of potential outcomes are possible. Interestingly, we saw strong relationships between the outcomes observed during agar coculture and those from the hydroponic root assay. Of the 10 pseudomonad isolates interrogated on roots, the strains exhibiting the strongest inhibition of L. monocytogenes during agar coculture inhibited L. monocytogenes both on and off the plant root, while those pseudomonads that only moderately inhibited L. monocytogenes during agar coculture were more likely to demonstrate root colonization-specific effects in our hydroponic assay. Thus, in spite of extensive strain-level variation, we observed some phenotypic outcomes that were consistent across assay formats, indicating that monitoring colony-level antagonism using agar-based assays is a useful tool to identify bacterial coculture partners able to impede L. monocytogenes plant root colonization.

In addition to uncovering specific microbial interactions that altered L. monocytogenes’ ability to associate with plant roots, we also wanted to understand the ecologically important question (75, 76) of how the order of addition impacts bacterial succession and survival (77, 78). The order of addition has been demonstrated to impact the overall composition of microbial communities (75, 79) and is relevant to future applied studies, since it is difficult to eradicate established L. monocytogenes biofilms from surfaces (12, 80, 81). We therefore tested whether any of these rhizobacteria (many of which were antagonistic when coinoculated with 10403S) could invade and reduce L. monocytogenes 10403S cell numbers when it was precolonized on roots. Our data support previous findings that L. monocytogenes is difficult to remove once it has become established on surfaces (80), with many of the strains that were able to antagonize 10403S during coinoculation not being capable of impacting the number of 10403S CFU/plant when it was preestablished there. These results demonstrate that the order of colonization is relevant to the ability of L. monocytogenes to establish itself on plant roots.

We did observe that the four strongest antagonists (from both the agar and coinoculation assays) were able to reduce 10403S burden on the plant root even when L. monocytogenes was preestablished there. Additionally, we determined that this ability to reduce L. monocytogenes’ association with roots is not contact dependent and that secreted products were enough to elicit antagonism against L. monocytogenes. It is notable that the one specific antagonist of 10403S plant attachment (P. protegens Pf-5, ES1010) was able to reduce the CFU/plant numbers of 11 different preestablished L. monocytogenes strains in a contact-independent manner. From the existing data, it is unclear whether this reduction is due to cell lysis, growth inhibition, or dissociation with the root surface. Regardless, the ability of Pf-5 to broadly impact L. monocytogenes strains highlights the potential use of this strain (or the bioactive compounds it secretes) to reduce L. monocytogenes colonization on crops or in food-processing facilities. P. protegens Pf-5 dedicates ∼6% of its genome to secondary metabolites, many of which are genes predicted to generate antibiotic and antifungal compounds (82). Additionally, Pf-5 is being developed as a plant-growth-promoting rhizobacterium, an organism that protects plants from pathogens as well as promotes plant growth (82), making Pf-5 a potentially eco-friendly agricultural agent or bioadditive.

L. monocytogenes is prevalent in the environment; however, compared to the knowledge we have about L. monocytogenes’ role as a human pathogen, we know very little about the requirements for L. monocytogenes’ existence in natural settings. Overall, our study provides new data regarding how exogenous factors (such as other bacteria or environmental growth conditions) impact L. monocytogenes-plant-root interactions and demonstrate the need for continued research into these associations. As our global population and, thus, food crop production and consumption increase, disease-causing L. monocytogenes outbreaks from contaminated produce are likely to increase as well. Thus, understanding the influence of specific bacteria or communities of microbes on L. monocytogenes’ physiology and growth will be crucial for identifying mechanisms that can reduce the carriage of L. monocytogenes in pastures and on plant surfaces to reduce the possibility of foodborne illnesses.

## MATERIALS AND METHODS

### Bacterial strains and growth conditions.

All bacterial isolates and strains (Table 1) were stored at −80°C in 20% glycerol. Unless otherwise stated, the night before experiments were initiated, strains were plated on Lennox-lysogeny broth (10 g/liter tryptone, 5 g/liter NaCl, 5 g/liter yeast extract; RPI [Research Products International]) or, for Escherichia coli, on Miller-lysogeny broth (10 g/liter NaCl, 10 g/liter tryptone, 5 g/liter yeast extract) agar plates made with 1.5% (wt/vol) Bacto-Agar (BD Biosciences) and were grown overnight at 37°C. To begin liquid inoculations, several isolated bacterial colonies were suspended in LB to an OD_600_ of 0.5 and inoculated into the assay wells at a final concentration of OD_600_ of 0.02. All references to LB indicate the use of the lower-salt Lennox composition of LB. In instances where L. monocytogenes was grown at alternative temperatures prior to inoculation in the hydroponic assay, 10403S was grown at 4°C (for 3 weeks), RT (for 3 days), 30°C (2 days), and 37°C (24 h).

### Plasmid and conjugation.

E. coli SM10 (83) carrying pHPL3-mcherry (constructed in reference 84) was conjugated with L. monocytogenes 10403S as previously described (85, 86). For maintaining the plasmid in E. coli and selecting/maintaining the plasmid in L. monocytogenes, chloramphenicol was used at 10 μg/ml and 7.5 μg/ml, respectively.

### Sterilization and storage of seeds.

Arabidopsis thaliana ecotype (Columbia-0 or Col-0) seedlings were sterilized by chlorine gas exposure. Briefly, 3 ml of concentrated HCl was added to 100 ml of concentrated Clorox bleach in an enclosed container for 3 h inside a chemical fume hood. After sterilization, seeds were stored in Eppendorf’s in the dark at 4°C until use.

### Seedling growth on mesh.

Prepunched, 0.5-cm-diameter mesh circles (stretchable high-temperature PTFE plastic mesh; 1100t43; McMaster-Carr) were autoclaved and placed onto agar plates containing 0.5× Murashige-Skoog (MS) salts plus MES buffer (morpholineethanesulfonic acid buffer and ethylenediaminetetraacetic acid ferric sodium [NaFe-EDTA], constituting a stock solution at 5 ml/liter containing 5.57 g FeSO4·7H_2_O and 7.45 g of Na2·EDTA) at 50 mg/liter (RPI M70300-5.0); this is referred to as 0.5× MS throughout the paper. There was no sucrose added to plates or liquid cultures for assays using 0.5× MS + MES. Individual sterile seeds were placed on the mesh disks, and the plate was sealed using a gas-permeable tape (BS-25; Aeraseal Excel Scientific) and placed in a Conviron incubator for long-day conditions (16 h of light, 21°C daytime, 18°C at night) for 7 to 9 days.

### Hydroponic experiments.

After 7 to 9 days, germinated seedlings and their mesh were placed into 24-well Corning plates containing 1.6 ml of the indicated liquid medium and inoculated with bacteria at an OD_600_ of 0.02. The 24-well plate was covered with a plastic lid and left on the bench (static) at RT for 24 h unless otherwise stated. After 24 h, seedlings were removed from the mesh with sterilized forceps and placed into 1.5-ml Eppendorf tubes for sonication and quantification of root associated cells (number of CFU/plant). Sonicated samples were serially diluted and plated on 1× LB and 1× LB plates supplemented with 200 μg/ml streptomycin (to select for L. monocytogenes 10403S). For quantification of planktonic number of CFU/ml, medium was serially diluted from each well and plated to count CFU on 1× LB and 1× LB with 200 μg/ml streptomycin (to select for L. monocytogenes 10403S).

### Coinoculation experiments.

Experiments are performed as stated in “Hydroponic experiments,” above. Bacteria were individually suspended to an OD_600_ of 0.5 and inoculated into a single well (L. monocytogenes plus one other bacterium per well) each at an OD_600_ of 0.02 and incubated at RT under static conditions for 24 h.

### Invasion experiments.

L. monocytogenes colonies were resuspended to an OD_600_ of 0.5 in 1× LB, and seedlings were placed into this inoculum for 3 h, static, at RT. The L. monocytogenes-inoculated roots were then placed into wells where the invading bacteria were added at an OD_600_ of 0.02, incubated at RT, and kept static. Wells were treated as stated in “Hydroponic experiments,” above.

### Alternative hydroponic assay.

Seeds were sterilized and grown as stated above, except that for this assay two seeds were placed on a single mesh disk. Liquid cultures of L. monocytogenes strains were grown overnight in brain heart infusion (53286; Sigma) at 30°C in a Cel-Gro tissue culture rotator, speed 8 (1640Q; Thermo Scientific). L. monocytogenes strains were centrifuged and resuspended in 10 mM MgCl_2_, where the number of CFU was adjusted to ∼1 × 10^9^ CFU/ml. Seedlings were transferred to 24-well plates containing the bacterial suspensions. Plates were covered with a gas-permeable membrane and shaken at 150 rpm at room temperature for 3 h. Following incubation, seedlings were removed from the wells and transferred to a new 24-well plate containing 1 ml of fresh 10 mM MgCl_2_ for 10 min. After this incubation, seedlings were removed from the mesh using an aseptic technique and transferred to a new 24-well plate containing 1 ml of 10 mM MgCl_2_, and plates were sealed and sonicated. Homogenate was serially diluted and plated on 1× LB to determine the number of CFU/seedling.

### Sonication.

Colonized seedlings were removed from the mesh and placed into an Eppendorf tube with 500 μl of 0.5× MS and sonicated (Qsonica sonicator q700) at 15 A using a microtip for 12, 1-s pulses, with 1 s off between pulses. The sonicator tip was thoroughly cleaned between samples using 70% ethanol. Sonicated samples were serially diluted and plated onto LB plates supplemented with 200 μg/ml streptomycin to select for L. monocytogenes or onto LB plates to quantify the other rhizobacteria.

### Agar plate coculture.

Cells were scraped from overnight LB-agar plates and suspended to an OD_600_ of 0.5 and 1 μl spotted on LB-agar plates with 0.5 cm in the center of each colony. Initial screen plates were grown at 37°C and imaged at 48 h; plates in subsequent experiments were grown at 30°C and imaged at 48 h.

### CM.

Several colonies from overnight streak plates were suspended to an OD_600_ of 0.5 and added to the hydroponic assay wells at an OD_600_ of 0.02. The strains were grown with seedlings present in 0.5× MS + MES for 24 h. After 24 h, the liquid culture medium was collected and centrifuged for 3 min to pellet the bacteria, and the supernatant was filtered through a 0.22-μm filter to remove any cells. This sterile, cell-free conditioned medium (CM) was then added to wells as 1 part conditioned medium and 1 part fresh 0.5× MS + MES.

### Microscopy.

For microscopy imaging, L. monocytogenes 10403S(pHPL3-mCherry) was colonized, with and without ES620, as previously stated for the hydroponic experiment. Seedlings were removed from culture after 24 h and placed on a slide. To prevent the seedlings from being crushed, frame seals (Bio-Rad SLF0601) were used to create a space between slide and coverslip and filled with 0.5× MS to allow for better imaging. Images were taken with a Nikon Eclipse 80i compound fluorescence microscope.

### Statistics.

Statistical comparisons were performed using GraphPad Prism, version 10.0. Each dot represents a biological replicate (single seedling), and data were obtained from at least three independent experiments for all experiments. Error bars represent standard errors of the means. Statistics were first performed by comparing all groups using Kruskal-Wallis analysis of variance (ANOVA) when applicable. If the Kruskal-Wallis ANOVA demonstrated significance, then individual groups were compared using Mann-Whitney *t* tests. When only two groups were compared, we only used Mann-Whitney *t* tests. Asterisk denotes *P* values (*, *P < *0.05; **, *P < *0.01; ***, *P < *0.001).
