# Supplementary material for: Rhizobacteria Impact Colonization of Listeria monocytogenes on Arabidopsis thaliana Roots
Source: Appl Environ Microbiol. 2021 Nov 10;87(23):e01411-21. doi: 10.1128/AEM.01411-21 (PMC8579980; doi:10.1128/AEM.01411-21)
Supplement: Supplemental file 1 — Fig. S1 to S9, Table S1. Download aem.01411-21-s0001.pdf, PDF file, 6.6 MB [file aem.01411-21-s0001.pdf]

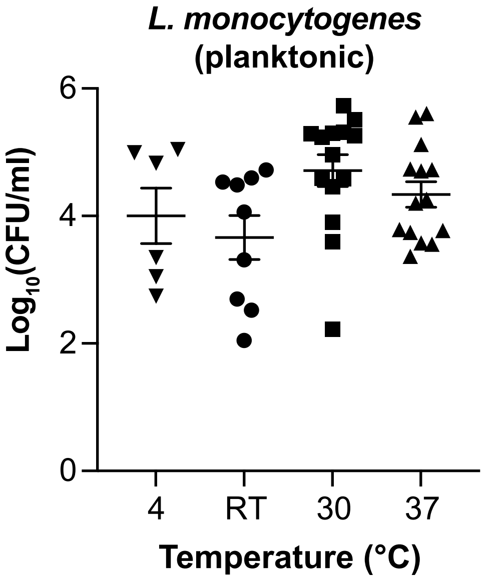

Supplemental Figure 1. Pre-Inoculation growth temperature does not alter *L. monocytogenes* 10403S CFU/ml without seedlings present. *L. monocytogenes* pre-grown at 4 °C, RT (20 – 22 °C), 30 °C, or 37 °C prior to inoculation were added to the hydroponic assay in 0.5X MS and incubated at RT for 24 h, after which CFU/ml liquid were determined. Kruskal-Wallis ANOVA and Mann-Whitney t-test were used for statistical comparisons. No significant differences were observed.

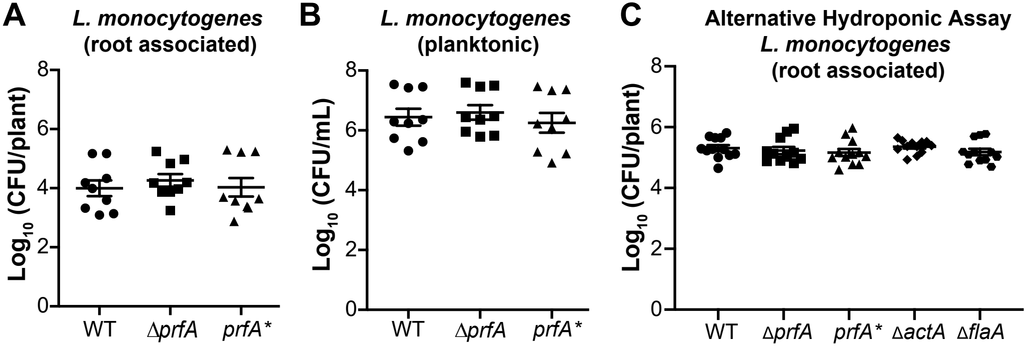

Supplemental Figure 2. *L. monocytogenes* 10403S *prfA*, *prfA, *flaA*, and *actA* mutants do not have altered root colonization compared to the 10403S parental strain.** (A, B) Using our conventional hydroponic assay, we compared root colonization of the parental 10403S to the *prfA* and *prfA** (constitutive) mutant strains. Samples were incubated at RT for 24 h before (A) CFU/plant and (B) CFU/ml liquid were determined from serial dilutions. (C) Using the modified hydroponic assay, we compared the CFU/plant of *L. monocytogenes* 10403S parental strain to the *prfA*, *prfA**, *flgA*, and *actA* mutants. After sonication, homogenate was serially diluted and plated on 1X LB to deter-mine CFU/plant. Error bars represent standard error of the mean. Statistical comparisons were performed using Kruskal-Wallis ANOVA. There were no statistically significant differences observed in any panel.

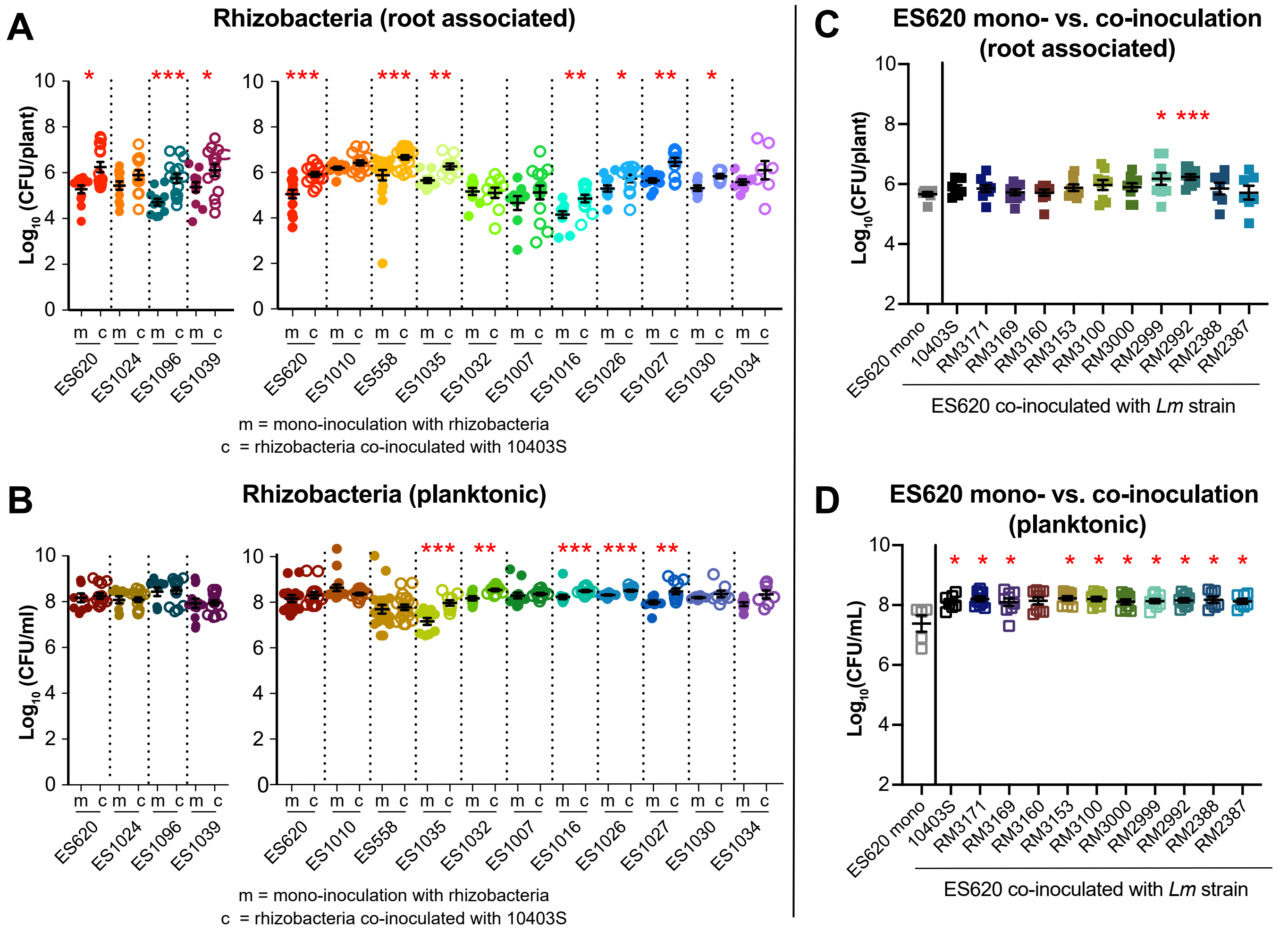

Supplemental Figure 3. Co-inoculation with *L. monocytogenes* enhances the root colonization and planktonic survival of many rhizobacteria. (A, B) *L. monocytogenes* 10403S was inoculated with multiple individual rhizobacteria by inoculating each bacterium at an OD_{600} = 0.02 in a 1:1 ratio and incubating with seedling roots for 24 h at RT. After incubation, seedlings were removed from the wells, homogenized, and serial dilutions were plated on 1X LB to calculate the (A) CFU/plant and (B) CFU/ml liquid. The rhizobacteria tested were *Pseudomonas simiae* WCS417r (ES620), *Arthrobacter nicotinovorans* (ES1024), *Curtobacterium oceanosedimentum* (ES1096), *Microbacterium oleivorans* (ES1039), *Burkholderia cenocepacia* (ES1010), *Pseudomonas fluorescens* Pf-5 (ES558), *Pseudomonas brassicacearum* (ES1035), *Pseudomonas sp.* (ES1032), *Pseudomonas fluorescens* BZ64 (ES1007), *Pseudomonas umsongensis* (ES1016), *Pseudomonas sp.* (ES1026), *Pseudomonas mandelii* (ES1027), *Pseudomonas sp.* (ES1030), and *Pseudomonas sp.* KD5 (ES1034). Rhizobacterial colonies were distinguished from *L. monocytogenes* 10403S by color and morphology. "m" indicates data obtained from mono-inoculations and "c" indicates data obtained during co-inoculations. The separated graphs in A and B represent independent series of experiments. (C, D) *P. simiae* WCS417r (ES620) was co-inoculated with 11 different *L. monocytogenes* strains (10403S and ten RM strains; see strain table) as described for panels A, B and the (C) CFU/plant and (D) CFU/ml liquid were determined. *P. simiae* (ES620) colonies were distinguished from *L. monocytogenes* colonies by color and morphology. In all panels, each dot represents a biological replicate (single seedling), and data were obtained from at least three biological independent experiments. Error bars represent the standard error of the mean. Statistics were performed via Kruskal-Wallis ANOVA and Mann-Whitney t-tests comparing coculture to monoculture CFU. Asterisk denotes *P* value (* = $P < 0.05$, ** = $P < 0.01$, *** = $P < 0.001$).

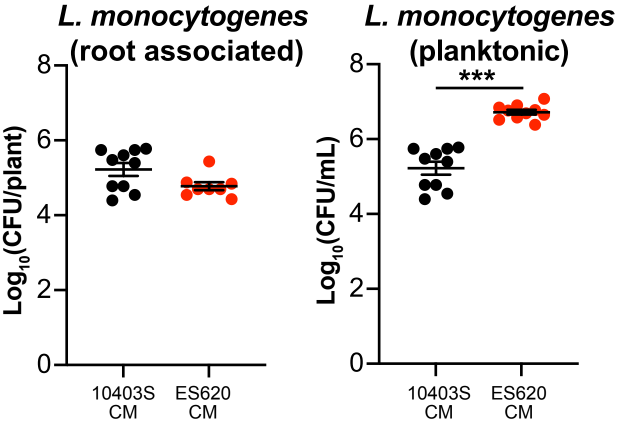

Supplemental Figure 4. Ability of ES620 (*Pseudomonas simiae* WCS417r) to enhance 10403S root association is contact dependent. Conditioned media (CM) was collected by centrifuging and filter sterilizing the liquid from bacteria grown with hydroponic plants after 24 h incubation at RT, then used fresh. CM from either *L. monocytogenes* strains (as a control) or ES620 were mixed at a 1:1 concentration with fresh media (0.5X MS:0.5X CM) in the assay. (A) *L. monocytogenes* CFU/plant and (B) CFU/ml were determined by serial dilutions on 1X LB. Statistics were performed using Mann-Whitney t-tests comparing the CFU/plant of *L. monocytogenes* grown in its own CM to *L. monocytogenes* grown in ES620 CM. Asterisks denote *P* value (* = $P < 0.05$, ** = $P < 0.01$, *** = $P < 0.001$). Dashed lines indicate the level of detection.

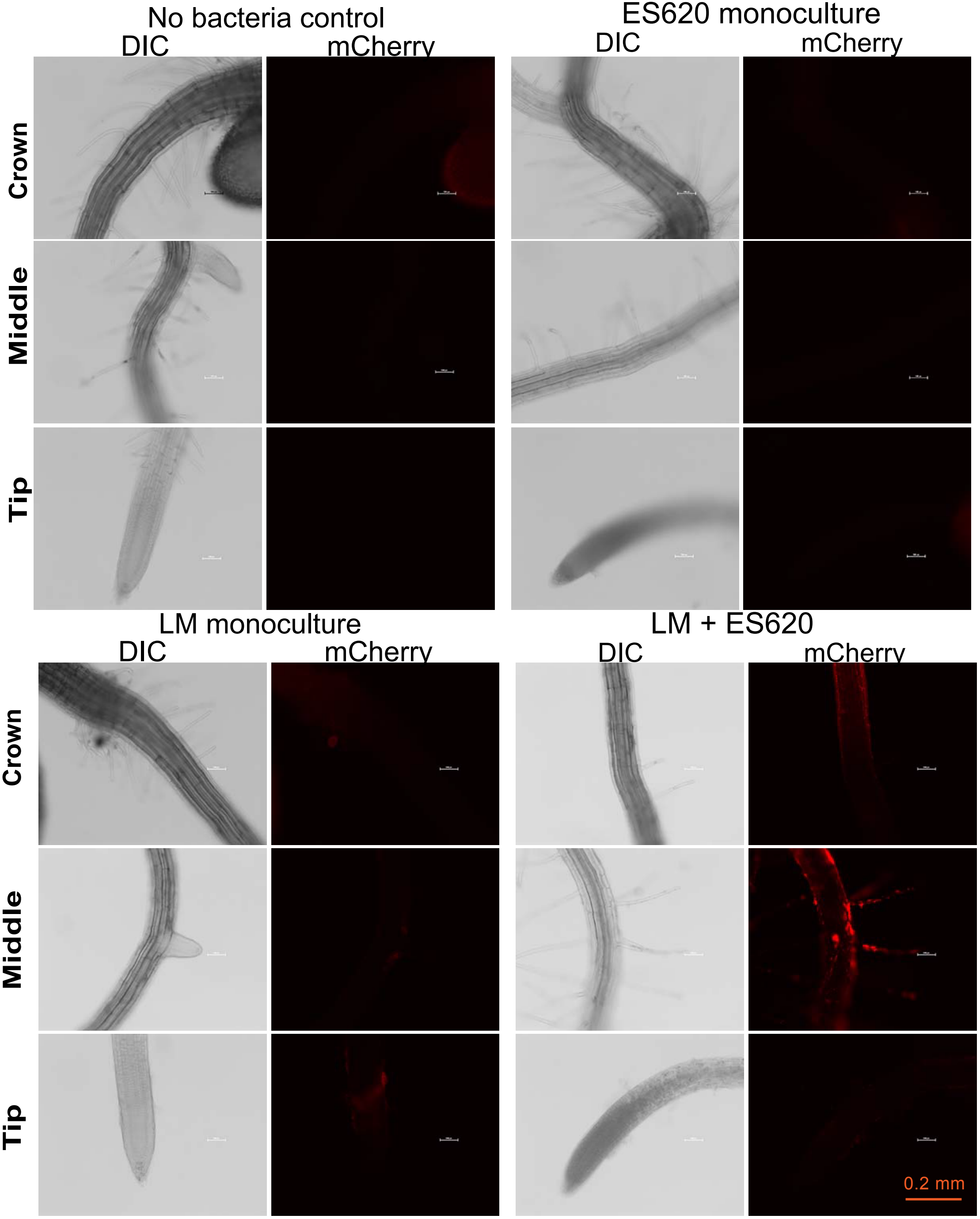

Supplemental Figure 5. ***Pseudomonas simiae*** **(ES620) enhances** ***L. monocytogenes*** **10403S root colonization primarily at the crown and middle of the seedling.** Representative images of *A. thaliana* seedling roots either not inoculated with bacteria (top left), inoculated with ES620 (*P. simiae*) alone (top right), *L. monocytogenes* 10403S(pHPL3) alone (bottom left) or ES620 and 10403S(pHPL3) in coculture (bottom right). For each set of panels, brightfield images are on the left, and corresponding fluorescence images (false-colored red, representing mCherry) are on the right. Scale bar = 0.2 mm.

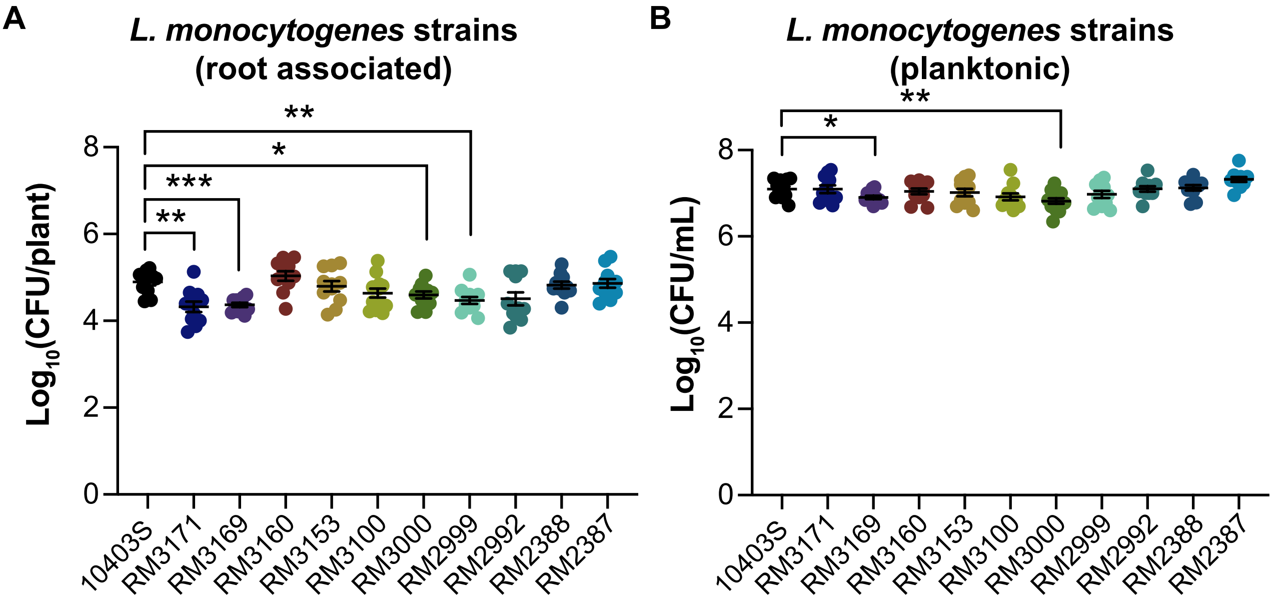

Supplemental Figure 6. A wide range of *L. monocytogenes* strains can robustly colonize *A. thaliana* roots. *L. monocytogenes* strains (10403S along with ten additional strains) were inoculated with in monoculture with *A. thaliana* seedlings for 24 h at RT. After incubation, seedlings were removed from the wells, homogenized, and serial dilutions plated on 1X LB to calculate the (A) CFU/plant and (B) CFU/ml liquid of each *L. monocytogenes* strain. Statistics were performed using Kruskal-Wallis ANOVA and Mann-Whitney t-tests comparing 10403S CFU levels to those of the other *L. monocytogenes* strains Asterisks denote *P* value (* = $P < 0.05$, ** = $P < 0.01$, *** = $P < 0.001$).

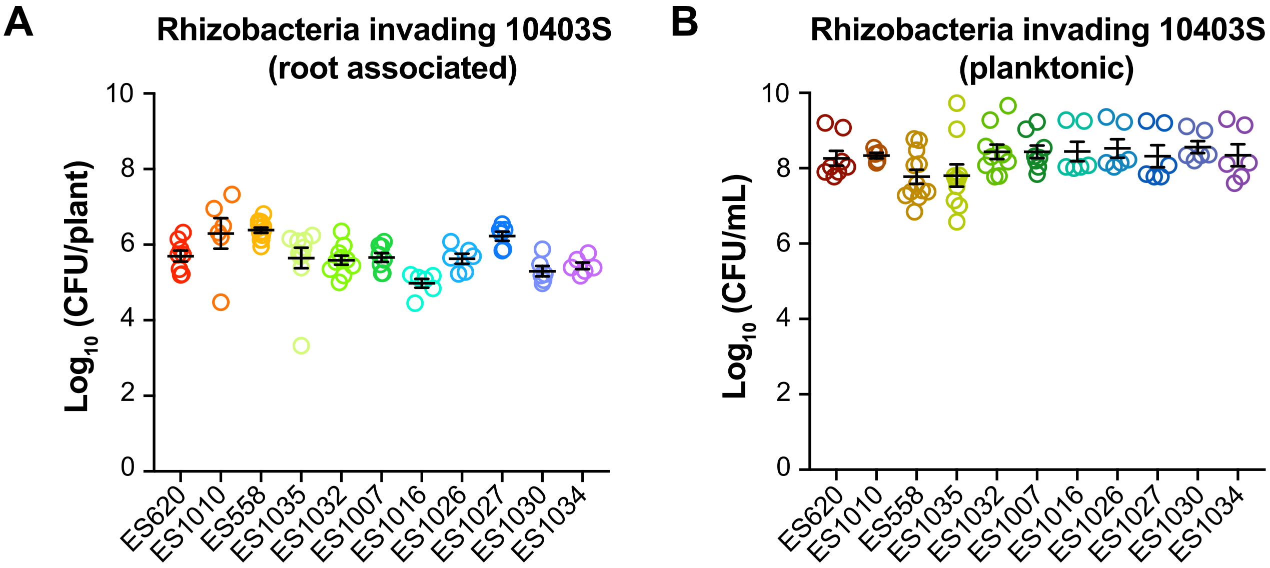

Supplemental Figure 7. Rhizobacteria are able to invade and colonize *A. thaliana* roots pre-associated with *L. monocytogenes* 10403S. In the invasion assay, *L. monocytogenes* 10403S was pre-loaded onto a seedling root (at ~10^5 CFU/plant) then transferred to wells with a single rhizobacterium at an OD_{600} = 0.02. After 24 h at RT, seedlings were removed, sonicated, and serially diluted on 1X LB for rhizobacteria to determine (A) CFU/plant and (B) CFU/ml liquid.

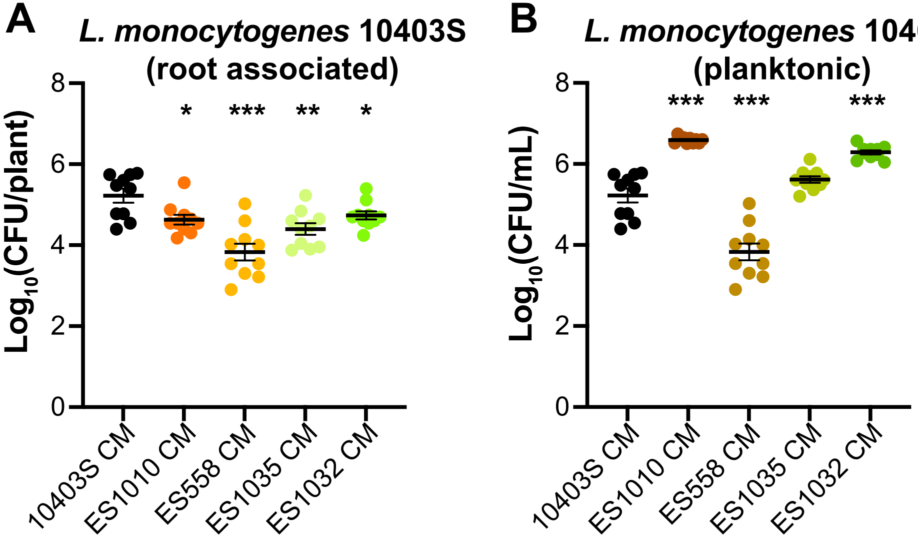

Supplemental Figure 8. The antagonism of ES1010, ES558, ES1035 and ES1032 towards *L. monocytogenes* 10403S is mediated by secreted products. Conditioned media (CM) was collected by centrifuging and filter sterilizing the liquid from bacteria grown with hydroponic plants after 24 h incubation at RT, then used fresh. CM from either *L. monocytogenes* 10403S (as a control) or rhizobacteria (*Burkholderia cenocepacia* (ES1010), *Pseudomonas fluorescens* Pf-5 (ES558), *Pseudomonas brassicacearum* (ES1035), *Pseudomonas sp*. (ES1032), were mixed at a 1:1 concentration with fresh MS (0.5X MS:0.5X CM) in the assay. (A) *L. monocytogenes* CFU/plant and (B) CFU/ml were determined by serially dilution on 1X LB. Statistics were performed using Mann-Whitney t-tests comparing CFU of *L. monocytogenes* grown in its own CM to *L. monocytogenes* grown in rhizobacteria CM. Asterisks denote *P* value (* = $P < 0.05$, ** = $P < 0.01$, *** = $P < 0.001$).

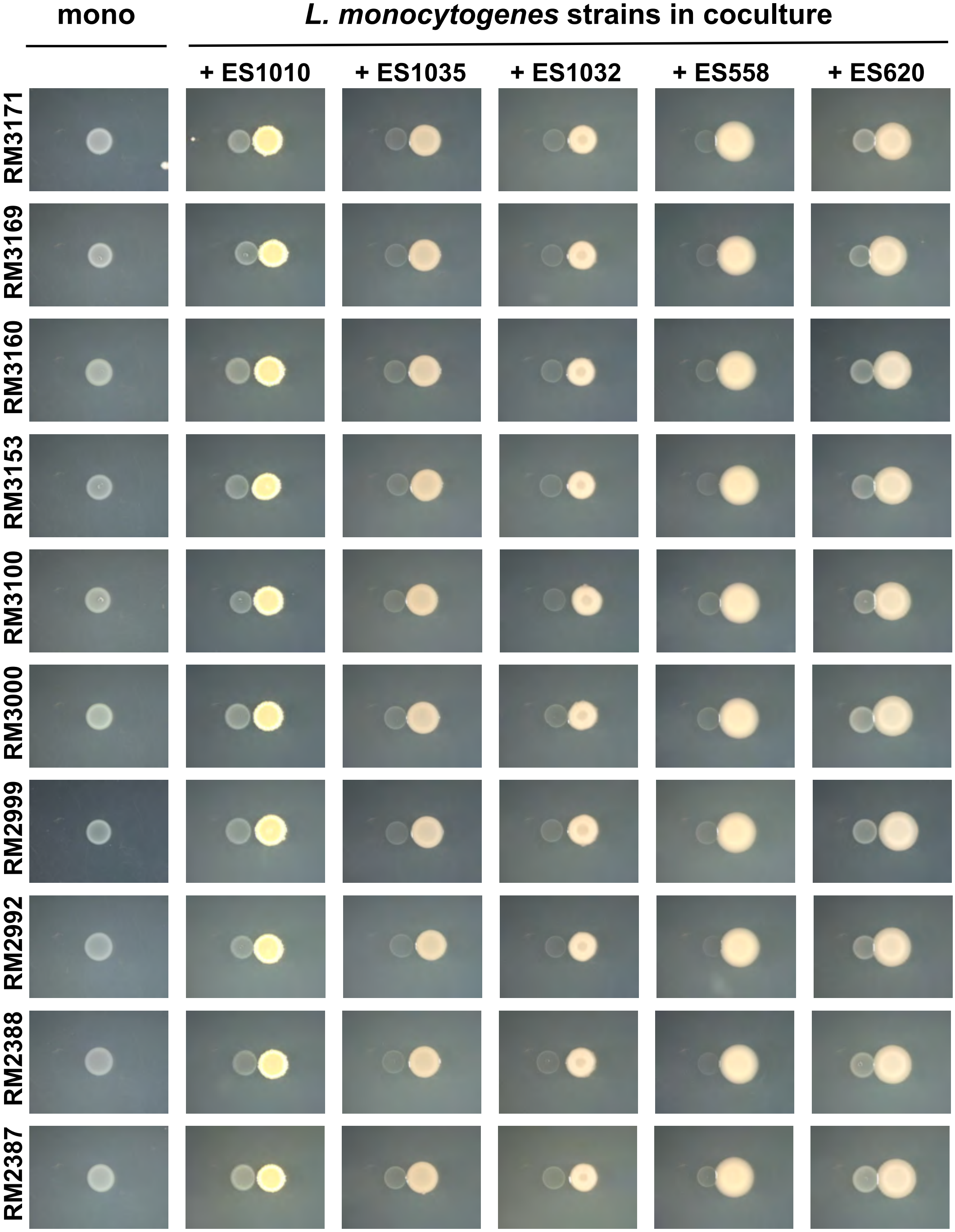

Supplemental Figure 9. *L. monocytogenes* strains grown in agar coculture with five rhizobacteria. *Burkholderia cenocepacia* (ES1010), *Pseudomonas brassicacearum* (ES1035), *Pseudomonas sp*. (ES1032), *Pseudomonas fluorescens* Pf-5 (ES558) and *Pseudomonas simiae* WCS417r (ES620) were cocultures with multiple *L. monocytogenes* (RM strains). Bacterial cells were scraped from plates of overnight growth, suspended in 1X LB to an OD_{600} = 0.5 and 2 µl of this bacterial suspension was pipetted onto 1X LB agar plates 0.5cm apart from the neighboring bacterial spot (based on center between spots). Plates were incubated at 30 ˚C for 48 h and then imaged. Antagonism was assessed by visually comparing the *L. monocytogenes* strains grown in monoculture to the colonies in coculture. A decrease in opacity (e.g., increase in transparency and translucency) was considered antagonism. Representative images are from a single experiment. Three independent experiments were performed with similar outcomes.

Supplemental Table 1: Interaction screening against *L. monocytogenes* 10403S

ES ID	Dangl ID	Genus	Antagonism in screen	Antagonism in follow up	Antagonism in hydroponic
ES558	NA	*Pseudomonas protegens* Pf-5	NA	XX	XXX
ES620	NA	*Pseudomonas WCS417r*	NA	-	+
ES965	CL002	*Ochrobactrum*	overgrown	NA	NA
ES966	CL004	*Brevundimonas*		NA	NA
ES967	CL010	*Microbacterium*		NA	NA
ES968	CL011	*Burkholderia*		NA	NA
ES969	CL012A	*Microbacterium*		NA	NA
ES970	CL013	*Bacillus*		NA	NA
ES971	CL014	*Variovorax*		NA	NA
ES972	CL017	*Curtobacterium*		NA	NA
ES974	CL019	*Bosea*		NA	NA
ES975	CL020	*Curtobacterium*		NA	NA
ES976	CL021	*Ralstonia*		NA	NA
ES978	CL028	*Arthrobacter*	X	NA	NA
ES979	CL032	*Agrobacterium*		NA	NA
ES980	CL033	*Phyllobacterium*		NA	NA
ES981	CL041	*Agrobacterium*		NA	NA
ES982	CL045	*Microbacterium*		NA	NA
ES983	CL052	*Paenibacillus*	overgrown	NA	NA
ES984	CL058	*Pseudomonas*	X	NA	NA
ES985	CL063	*Arthrobacter*		NA	NA
ES986	CL069	*Acinetobacter*		NA	NA
ES987	CL071	*Acinetobacter*		NA	NA
ES988	CL081	*Bacillus*		NA	NA
ES989	CL089	*Microbacterium*		NA	NA
ES991	CL096	*Bacillus*	overgrown	NA	NA
ES992	CL115	*Bacillus*	overgrown	NA	NA
ES994	CL125	*Methylobacterium*		NA	NA
ES995	CL126	*Methylobacterium*		NA	NA
ES996	CL127	*Microbacterium*		NA	NA
ES997	CL129	*Methylobacterium*		NA	NA
ES998	CL130	*Paenibacillus*	XXX	NA	NA
ES999	CL136	*Methylobacterium*		NA	NA
ES1000	CL140	*Microbacterium*		NA	NA
ES1001	CL141A	*Paenibacillus*	X	NA	NA

ES1002	CL143	*Methylobacterium*		NA	NA
ES1003	CL144	*Ralstonia*		NA	NA
ES1004	CL152	*Microbacterium*		NA	NA
ES1005	CL154	*Leifsonia*		NA	NA
ES1006	MF002	*Rhizobium*	X	NA	NA
ES1007	MF003	*Pseudomonas*	overgrown	-	X
ES1008	MF004	*Variovorax*		NA	NA
ES1009	MF005	*Bacillus*	overgrown	NA	NA
ES1010	MF006	*Burkholderia*	XX	NA	XXX
ES1011	MF007	*Burkholderia*	XX	NA	NA
ES1012	MF008	*Chryseobacterium*		NA	NA
ES1013	MF009	*Arthrobacter*		NA	NA
ES1014	MF010	*Agrobacterium*		NA	NA
ES1015	MF011	*Microbacterium*		NA	NA
ES1016	MF020	*Pseudomonas*		-	X
ES1017	MF021	*Leifsonia*		NA	NA
ES1018	MF022	*Luteibacter*		NA	NA
ES1019	MF023	*Rhodococcus*		NA	NA
ES1020	MF025	*Ralstonia*		NA	NA
ES1021	MF026	*Arthrobacter*		NA	NA
ES1022	MF027	*Bacillus*		NA	NA
ES1023	MF029	*Rhodococcus*		NA	NA
ES1024	MF031	*Arthrobacter*	X	NA	-
ES1025	MF033	*Agrobacterium*		NA	NA
ES1026	MF035	*Pseudomonas*		X	X
ES1027	MF036	*Pseudomonas*	XX	XX	X
ES1028	MF040	*Flavobacterium*		NA	NA
ES1030	MF045	*Pseudomonas*		XX	X
ES1032	MF048	*Pseudomonas*	XXX	XXX	XXX
ES1033	MF049	*Arthrobacter*		NA	NA
ES1034	MF048	*Pseudomonas*		X	X
ES1035	MF051	*Pseudomonas*	XXX	XXX	XX
ES1036	MF057	*Rhizobium*		NA	NA
ES1039	MF077	*Microbacterium*		NA	-
ES1040	MF079	*Dyella*		NA	NA
ES1041	MF088	*Mycobacterium*		NA	NA
ES1042	MF092	*Stenotrophomonas*		NA	NA
ES1043	MF095	*Bacillus*	overgrown	NA	NA

ES1044	MF098A	*Leifsonia*		NA	NA
ES1047	MF106	*Bacillus*		NA	NA
ES1048	MF109	*Leifsonia*		NA	NA
ES1049	MF110	*Variovorax*		NA	NA
ES1050	MF111	*Methylobacterium*		NA	NA
ES1051	MF112	*Bacillus*	overgrown	NA	NA
ES1052	MF113	*Pseudomonas*	X	NA	NA
ES1053	MF114	*Rhodococcus*		NA	NA
ES1055	MF123	*Bacillus*		NA	NA
ES1058	MF135	*Arthrobacter*		NA	NA
ES1062	MF157	*Leifsonia*		NA	NA
ES1063	MF160	*Variovorax*		NA	NA
ES1064	MF161	*Arthrobacter*		NA	NA
ES1067	MF166A	*Bacillus*	overgrown	NA	NA
ES1070	MF178	*Dyella*		NA	NA
ES1071	MF181	*Paenibacillus*	XXX	NA	NA
ES1072	MF190	*Methylobacterium*		NA	NA
ES1073	MF196	*Bacillus*	overgrow	NA	NA
ES1074	MF212	*Bacillus*	overgrown	NA	NA
ES1075	MF215	*Bacillus*		NA	NA
ES1076	MF217	*Paenibacillus*	overgrown	NA	NA
ES1077	MF220A	*Sphingomonas*		NA	NA
ES1078	MF224	*Agrobacterium*		NA	NA
ES1079	MF231	*Arthrobacter*	X	NA	NA
ES1080	MF254	*Arthrobacter*		NA	NA
ES1081	MF261	*Microbacterium (IMG wrongly says Mycobacterium)*		NA	NA
ES1082	MF267	*Mycobacterium*		NA	NA
ES1083	MF273	*Terracoccus*		NA	NA
ES1084	MF275	*Methylobacterium*		NA	NA
ES1085	MF278	*Variovorax*		NA	NA
ES1087	MF283	*Mycobacterium*		NA	NA
ES1088	MF285	*Methylobacterium*		NA	NA
ES1092	MF300	*Methylobacterium*		NA	NA
ES1093	MF302	*Phyllobacterium*		NA	NA
ES1095	MF312A	*Chryseobacterium*		NA	NA
ES1096	MF314	*Curtobacterium*		NA	-
ES1097	MF322	*Bacillus*	overgrown	NA	NA

ES1098	MF327	*Promicromonospora*		NA	NA
ES1105	MF349	*Variovorax*		NA	NA
ES1106	MF350	*Variovorax*		NA	NA
ES1109	MF360	*Mycobacterium*		NA	NA
ES1110	MF362	*Arthrobacter*		NA	NA
ES1111	MF363	*Rhodococcus*		NA	NA
ES1113	MF366	*Luteibacter*		NA	NA
ES1114	MF370	*Ochrobactrum*	X	NA	NA
ES1115	MF374	*Brevundimonas*		NA	NA
ES1116	MF375	*Variovorax*		NA	NA
ES1117	MF376	*Burkholderia*	X	NA	NA
ES1118	MF384	*Burkholderia*		NA	NA
ES1119	MF395	*Pseudomonas*	XXX	NA	NA
ES1120	MF397	*Pseudomonas*	XXX	NA	NA
ES1121	MF467	*Leifsonia*		NA	NA
ES1123	MF496	*Paenibacillus*		NA	NA

Supplemental Table 1: ES ID pertains to the strain designation within the Shank Lab Strain Collection, while Dangl ID reflects the ID pertaining to Dangl Strain Collection and NCBI identification (1, 2). The level of antagonism is depicted by an X (minor), XX (moderate), XXX (high level), while a “-” indicates no antagonism was observed and “+” indicates better growth of *L. monocytogenes.* Overgrown indicates that the rhizobacteria overgrew the *L. monocytogenes* colony and it was impossible to determine whether antagonism was present. If an organism was not used in a given assay it is listed as NA.

SUPPLEMENTAL REFERENCES

1. Lebeis SL, Paredes SH, Lundberg DS, Breakfield N, Gehring J, McDonald M, Malfatti S, Glavina del Rio T, Jones CD, Tringe SG, Dangl JL. 2015. Salicylic acid modulates colonization of the root microbiome by specific bacterial taxa. Science 349:860-864.
2. Lundberg DS LS, Paredes SH, Yourstone S, Gehring J, Malfatti S, Tremblay J, Engelbrektson A, Kunin V, Del Rio TG, Edgar RC, Eickhorst T, Ley RE, Hugenholtz P, Tringe SG, Dangl JL. 2012. Defining the core Arabidopsis thaliana root microbiome. Nature 488:86-90.
